# Radical *en bloc* peritonectomy in advanced ovarian cancer

**DOI:** 10.3332/ecancer.2018.808

**Published:** 2018-02-08

**Authors:** Víctor Lago, Santiago Domingo, Luis Matute, Pablo Padilla-Iserte, Marta Gurrea

**Affiliations:** 1 Department of Gynecologic Oncology, University Hospital La Fe, Valencia 46026, Spain; 2 orcid.org/0000-0002-2971-1899

**Keywords:** advanced ovarian cancer, debulking surgery, radical peritonectomy

## Abstract

In order to reach cytoreduction in advanced ovarian cancer, peritonectomy and diaphragmatic stripping are procedures required to remove the disease in the upper abdomen. Diaphragm involvement is estimated in up to 40% of cases. Nevertheless, in some of these patients, the tumour volume may constitute a limitation of the technique due to the association with abdominal wall involvement, bulky tumour at the Morrison’s pouch or liver infiltration. Extensive upper abdominal procedures should represent a basic resource for the gynaecologic oncologist in order to reach an optimal cytoreduction. A radical peritonectomy with en bloc resection for treating advanced ovarian cancer with extensive widespread diaphragmatic peritoneal carcinomatosis is showed in this surgical film.

## Objective

In patients with advanced ovarian cancer, peritoneal carcinomatosis is commonly reported. Peritonectomy and diaphragmatic stripping are the elective procedures chosen to remove the disease in the upper abdomen at the diaphragm [1]. Nevertheless, in some cases the tumour volume may represent a limitation of the technique due to abdominal wall involvement, bulky tumour at the Morrison’s pouch or liver infiltration [2]. A radical peritonectomy with en bloc resection for treating advanced ovarian cancer with peritoneal carcinomatosis involving abdominal wall, liver and bulky tumour at Morrison’s pouch is shown in a surgical film (Video 1).

## Methods

A patient diagnosed with a high-grade serous carcinoma of the ovary is presented. Computed tomography demonstrated subdiaphragmatic implants, omental cake, and bilateral pelvic mass infiltrating the uterus, adnexa, cul-de-sac and pelvic peritoneum. Primary debulking surgery was considered after laparoscopic assessment. Peritoneal carcinomatosis infiltrating abdominal wall and liver was found. Bulky tumour at Morrison’s pouch was also present. A radical peritonectomy with en bloc resection of the described locations was performed.

## Results

After modified posterior pelvic exenteration, cholecystectomy, splenectomy and radical omentectomy were performed, the upper abdomen was adequately exposed. From beyond the upper limb, parietal peritoneum was dissected from the subdiaphragmatic, paracolic areas and Morrison’s pouch. The tumour present at the right abdominal wall and liver parenchyma was resected en bloc with the parietal peritoneum (Video 1).

## Discussion

At the upper abdomen, tumour is commonly present in advanced ovarian cancer. Diaphragm involvement is estimated in about 40% of the patients with advanced ovarian cancer [3]. Diaphragm peritonectomy procedure is related with an acceptable morbidity rate [4]. As a result, extensive upper abdominal techniques should represent a basic resource of the gynaecologic oncologist to achieve an optimal cytoreduction.

## Conclusions

Radical peritonectomy with en bloc abdominal wall and liver resection is a feasible procedure for removing metastasis in advanced ovarian cancer patients in order to reach an optimal cytoreduction.

## Figures and Tables

**Video 1.**
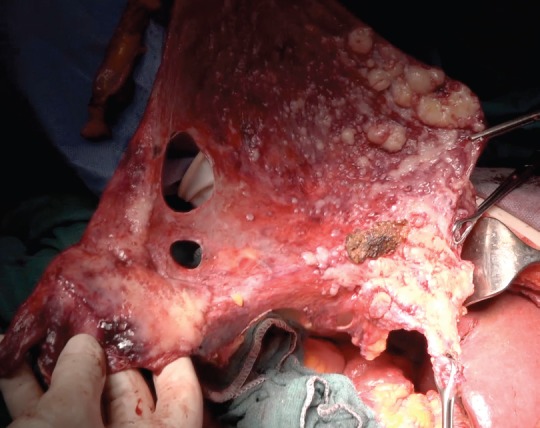
A radical peritonectomy procedure is shown in this video, in which a tumour present at diaphragm peritoneum, abdominal wall and liver parenchima was en bloc resected. To view this video, click here: https://ecancer.org/journal/12/808-Radical-en-bloc-peritonectomy-in-advanced-ovarian-cancer.php
